# Genetic relationships between systemic lupus erythematosus and a positive antinuclear antibody test in the absence of autoimmune disease

**DOI:** 10.1136/lupus-2024-001476

**Published:** 2025-06-12

**Authors:** Atlas Khan, Gul Karakoc, Ge Liu, Jacy Zanussi, Nancy J Olsen, Mingjian Shi, Nancy J Cox, Jonathan Mosley, Charles Michael Stein, Krysztof Kiryluk, Wei-Qi Wei, Frank Mentch, Scott Hebbring, James Linneman, Vivian Kawai

**Affiliations:** 1 Columbia University Vagelos College of Physicians and Surgeons, New York city, New York, USA; 2 Department of Medicine, Vanderbilt University Medical Center, Nashville, Tennessee, USA; 3 Department of Medicine, Penn State Health Milton S Hershey Medical Center, Hershey, Pennsylvania, USA; 4 Department of Medicine, University of Texas Southwestern Medical Center, Dallas, Texas, USA; 5 The Children’s Hospital of Philadelphia Center for Applied Genomics, Philadelphia, Pennsylvania, USA; 6 Marshfield Clinic Research Institute, Marshfield, Wisconsin, USA; 7 Department of Medicine, The University of Alabama at Birmingham, Birmingham, Alabama, USA

**Keywords:** autoantibodies, lupus erythematosus, systemic, polymorphism, genetic, risk factors

## Abstract

**Objective:**

We defined the genetic factors associated with a positive ANA test (ANA+) in the absence of autoimmune disease and tested the association with SLE.

**Methods:**

Using a case-control design, we performed a genome-wide association study (GWAS) in individuals of European ancestry without an autoimmune disease who had ANA tested as part of clinical care from DNA biobanks linked to de-identified electronic medical records: BioVU and Electronic Medical Records and Genomics. GWAS results were meta-analysed and single nucleotide polymorphism (SNP) heritability was calculated. A polygenic risk score (PRS) for ANA+ and for SLE was constructed and compared in patients with SLE, ANA+ and ANA negative (ANA−) individuals without autoimmune disease and general controls who never had ANA testing performed.

**Results:**

A total of 7287 individuals of European ancestry were included in the meta-analyses (2169 ANA+ and 5118 ANA−); an SNP upstream of the *TSBP1* in the HLA locus (rs1967688) was associated with ANA+ (p=4.84×10^−8^). SNP heritability for ANA+ was low (h^2^_SNP_= 0.04), and the PRS for ANA+ was not significantly different in ANA+ and ANA− individuals. In contrast, the PRS for SLE was significantly higher in SLE compared with ANA+ individuals (p<2.2×10^−16^) but did not differ among ANA+, ANA− and general control groups (p=0.17).

**Conclusions:**

ANA+ occurring in the absence of autoimmune disease has a genetic association with the *HLA* region, but overall heritability is low. In addition, few SLE-associated SNPs were associated with ANA+, and the PRS for SLE was not associated with ANA+, indicating limited genetic overlap.

**WHAT IS ALREADY KNOWN ON THIS TOPIC**

- A positive ANA test is present in almost all patients with SLE, but it also can occur in up to 20% of individuals in the general population.
- A previous study in Japanese individuals suggested that genetic variation in the HLA region (in linkage with HLA-DRB1*0405) was associated with a positive ANA.

**WHAT THIS STUDY ADDS**

- This is the largest study to define the genetics of a positive ANA test in 7287 individuals without an autoimmune disorder of European ancestry (2169 individuals with a positive test and 5118 with a negative test).
- We rigorously followed current recommendations for ANA titre thresholds and included only individuals with an ANA titre ≥1:80 and those with a negative test to avoid misclassification.
- The SNP-based heritability of ANA titre of ≥1:80 was low, and the genome-wide association study found a single significant association upstream of the *TSBP1* in the HLA locus.
- We found that a polygenetic risk score (PRS) for SLE discriminated between individuals with and without SLE, but it did not discriminate between individuals with and without a positive ANA test.

**HOW THIS STUDY MIGHT AFFECT RESEARCH, PRACTICE OR POLICY**

- Our findings suggest that in individuals without autoimmune disease who have a positive ANA test: (1) non-genetic factors may be more important than genetic factors and (2) there is little genetic overlap with SLE.
- Additionally, a PRS for SLE could discriminate between the presence or absence of SLE in people with a positive ANA test.

## Introduction

 ANAs are a diverse group of antibodies that are routinely measured as part of the clinical evaluation for the diagnosis of SLE and several other autoimmune disorders. A positive ANA test at a titre of 1:80 or greater is a requirement to meet the classification criteria for SLE;^1^ however, approximately 12%–20% of the general population have a positive ANA in the absence of an autoimmune disorder, and 2% have high titres.^2^

In some autoimmune diseases such as SLE, ANAs play a role in disease pathogenesis through the deposition of immune complexes in tissues, promotion of cytokine production, formation of neutrophil extracellular traps and cross-reactivity with different cellular antigens.^3^ The role of ANAs occurring in the absence of autoimmune disease is, however, unclear. Some studies have suggested associations with atherosclerosis,^4^ cardiovascular events,^5^ several types of cancer^6^,^8^ and all-cause mortality.^9^ We previously found that a positive ANA in the absence of autoimmune disease was associated with an increased risk of Raynaud’s syndrome and alveolar/perialveolar-related pneumopathies.^10^ Moreover, the immunological profile of people with a positive ANA without autoimmune disease is altered and characterised by elevated levels of pro-inflammatory mediators, antibody production and upregulation of genes involved in autoimmune diseases.^11 12^

These observations suggest that people without autoimmune diseases who have a positive ANA test may share some of the immunological dysregulation characteristics of SLE and, thus, may have a similar genetic predisposition. Accordingly, comparing the genetic predisposition with SLE and a positive ANA in the absence of autoimmune disease could provide insights into shared biology. The genetics of SLE are well-characterised^13 14^ and twin studies have shown that ANA production in SLE has a genetic component;^15^ however, the genetic architecture of ANA production is not well-defined and genome-wide association studies (GWAS) for positive ANA have not been performed in populations of European ancestry.

We hypothesised that individuals with a positive ANA in the absence of autoimmune disease and SLE would only partially overlap genetic architectures. Thus, defining the architecture of ANA positivity could improve our understanding of the biology of these diagnoses and identify genetic factors that discriminate individuals at risk for SLE from those with a positive ANA. To define the genetic architecture of a positive ANA without an autoimmune disease, we performed a GWAS using the current recommended titre threshold for ANA positivity (≥1:80)^1^ and compared the SNPs associations with those in patients with SLE to better understand their genetic relationships.

## Materials and methods

### Study population

We used BioVU, the VUMC DNA biobank linked to a de-identified electronic health record (EHR) system,^16^ and the Electronic Medical Records and Genomics (eMERGE) network consortium.^17 18^

**Figure 1:**
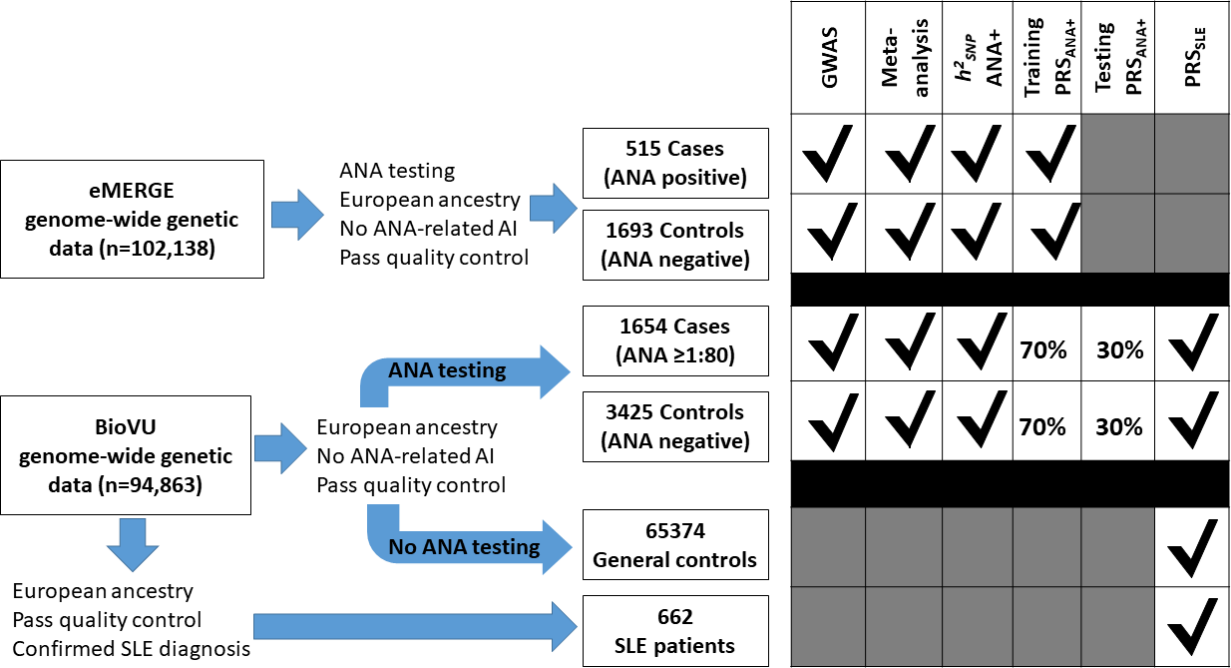
Flow chart showing the study design. Eligible individuals with an ANA test as part of their clinical care from Electronic Medical Records and Genomics (eMERGE) and BioVU were included in the site-specific genome-wide association studies (GWAS) and in the meta-analysis. Meta-analysis summary results were used to estimate single nucleotide polymorphism (SNP)-based heritability for positive ANA. In addition, to construct a polygenic risk score for positive ANA (PRS_ANA+_), we included data from 70% of ANA tested eligible individuals from BioVU and all individuals from eMERGE in the training set. The remaining 30% of ANA tested individuals from BioVU (testing set) were used to test the PRS_ANA+_. To study the performance of a polygenic risk score for SLE (PRS_SLE_), we estimated the PRS_SLE_ in ANA tested individuals, general controls and patients with SLE from BioVU. AI, autoimmune disease.

BioVU cohort: in BioVU, we selected individuals with genome-wide genotype data available on the Illumina Infinium Multi-Ethnic Genotyping Array (MEGA^EX^) platform that had an ANA test done as part of their clinical care. We excluded individuals: (a) with at least one diagnostic code for SLE or common autoimmune diseases known to be associated with a positive (online supplemental table S1), (b) without an ANA titre reported or had a titre of 1:40, (c) who were also included in the eMERGE cohort and/or (d) from other ancestries except European descent. We defined four mutually exclusive groups (figure 1): (a) *ANA positive (ANA+) cases* included individuals without an autoimmune disease with an ANA titre of 1:80 or higher. For individuals with more than one ANA test recorded in their EHR, the highest ANA titre was selected to determine eligibility; (b) *ANA negative (ANA−) controls* included individuals without an autoimmune disease who had only negative ANA results in the EHR; (c) *patients with SLE* included those diagnosed with SLE by a rheumatologist, nephrologist or dermatologist and (d) *general controls* included genotyped individuals who did not have a diagnosis of autoimmune disease and were not included in any of the previous groups. To define SLE cases, extensive manual chart review of the EHRs of individuals with at least one diagnostic code for SLE was performed by two physicians (VK and JG) and one rheumatologist (CMS) to ascertain the diagnosis of SLE.

eMERGE cohort: the eMERGE network consortium consists of 12 medical centres with EHRs linked to genome-wide genotype data for 102 138 individuals.^17 18^ Similar to the BioVU cohort, individuals with an ANA test performed as part of their clinical care were selected, and individuals with a diagnostic code for a common autoimmune disease, as well as those from other ancestries except European, were excluded. Individuals with a positive ANA test and those with only negative results were considered *ANA+ cases* and *ANA− controls*, respectively.

### Genotyping and imputation

BioVU cohort: genotyping was performed by the Vanderbilt Technologies for Advance Genomics (VANTAGE) according to standard protocols on the MEGA^EX^ platform. Quality control (QC) analyses used PLINK V.1.9 beta.^19^ Samples were excluded if there was a missingness rate >4%, discordance between genetically determined and reported sex or duplicated and related individuals.^20^ Principal components (PCs) were calculated using the SNPRelate package.^21^ PCs for ancestry were calculated using common variants (minor allele frequency (MAF) >1%) with high variant call rate (>98%), excluding SNPs that deviated from Hardy-Weinberg equilibrium (HWE, p<1×10^−6^), and SNPs in linkage disequilibrium (LD). We restricted our analysis to a homogeneous population of European descendant individuals using PCs and the HapMap populations as reference to include any subject within ±4 SD of the median values for European ancestry. To increase genomic coverage, genotype imputation was performed using Michigan Imputation Server using Haplotype Reference Consortium (HRC) V.r1.1 reference panel in genome build 37 (hg19).^22^ Imputed data were filtered for a sample missingness rate <2% and SNP missingness rate <4%. For association analyses, we filtered (1) poorly imputed variants with r^2^ value of <0.7, (2) MAF <5% and (3) variants with MAF different from the HRC V.r1.1 reference panel (MAF differences >0.3). SNP deviation from HWE and multiallelic SNPs and structural variants were also excluded.

eMERGE cohort: the genotyping and imputation of the eMERGE cohort have been described in detail.^23 24^ Briefly, the mimimac3 missing variant imputation model with genome-wide imputation was implemented using the HRC V.r1.1 reference for each genotyping platform in a separate batch. After imputation, all the 81 imputed batches were merged based on position using bcftools (http://researchcomputing.syr.edu/bcftools/). The QC filters required a marker to have a minor allele frequency and imputation quality r^2^>0.8 in at least 75% of 81 imputation batches. PC analysis for each cohort was performed using FlashPCA2. KING software was used to identify cryptically related subjects, and one individual per related pair with a second degree or higher relatedness was removed.^25^

### Statistical analysis

The analyses were restricted to individuals of European ancestry. Figure 1 described the cohorts and analyses performed, which included the following:

- GWAS were performed separately in BioVU and eMERGE. In both cohorts, *ANA+ cases* and *ANA− controls* were compared using logistic regression and an additive model in Plink V.2.0. A threshold of p≤5×10^−8^ and a p≤1×10^−5^ defined significant and nominal associations, respectively. Analyses were adjusted for sex, age and the first five PCs to reduce any potential bias from population stratification.
- Random effect meta-analyses were performed to combine GWAS summary results from BioVU and eMERGE. Cochran’s Q and I^2^ statistics were used to assess between-study allelic effect size heterogeneity. Weighted z-scores and weighted p values were calculated based on study sample sizes.^26^ Manhattan plots and quantile-quantile plots were constructed using R V.4.2.3. Expression quantitative loci (eQTL) databases, including GTEx V.810 and Blood eQTL,^27^ were explored to determine whether significant SNPs affect the expression of cis-genes in blood.
- SNP-based heritability (h^2^_SNP_), proportion of ANA+ variability explained by common variants, was estimated using Linkage Disequilibrium Score Regression (LDSC) V.1.0.1,^28^ and summary results from the meta-analysis.
- Genetic relationship between ANA+ and SLE using polygenetic risk score (PRS) approach: a PRS for ANA+ (PRS_ANA+_) was constructed by randomly dividing *ANA+ cases* and *ANA− controls* in BioVU into a training (70%) and a testing (30%) set. Summary-level data from a meta-analysis that included GWAS results in the training sample and in eMERGE was used to construct the PRS_ANA+_ using a Bayesian framework with continuous shrinkage.^29^ The PRS_ANA+_ was then validated in the testing set. In addition, summary statistics from the largest GWAS analysis of SLE in a European population^13^ were extracted to determine: (i) the association between SLE-associated SNPs (p≤5×10^−8^) and ANA+, (ii) the genetic correlation between ANA+ and SLE using LDSC V.1.0.1^30^ and (iii) if genetic susceptibility for SLE quantified as a PRS using the Bayesian framework (PRS_SLE_)^29^ could differentiate patients with SLE. The standardised PRS_SLE_ was compared among *ANA+ cases*, *ANA− controls*, *patients with SLE* and *general controls* in the BioVU cohort.

For the GWAS and meta-analysis, associations with a p≤5×10^−8^ were considered significant. To define the association between SLE-associated SNPs and ANA+, a Bonferroni corrected p value was used (0.05/number of SNPs tested); for all other analyses, a p value <0.05 was considered statistically significant.

## Results

### Study population

In BioVU, there were 12 639 individuals with genotype data available and an ANA test done as part of their clinical care. We excluded individuals with at least one diagnostic code for SLE or common autoimmune diseases known to be associated with a positive ANA (n=4424, online supplemental table S1), those without an ANA titre reported or with a titre of 1:40 (n=863), those who were also included in the eMERGE cohort (n=801) and those from ancestries other than European (n=1177). Characteristics of the eligible ANA individuals are shown in online supplemental table S2. In addition, there were 65 374 *general controls* and 662 *patients with SLE*.

In eMERGE, individuals with an ANA test performed as part of their clinical care (n=4896) were selected, and individuals with a diagnostic code for a common autoimmune disease (n=2688) as well as those from ancestries other than European (n=1495) were excluded.

### ANA+ GWAS and meta-analysis

**Figure 2:**
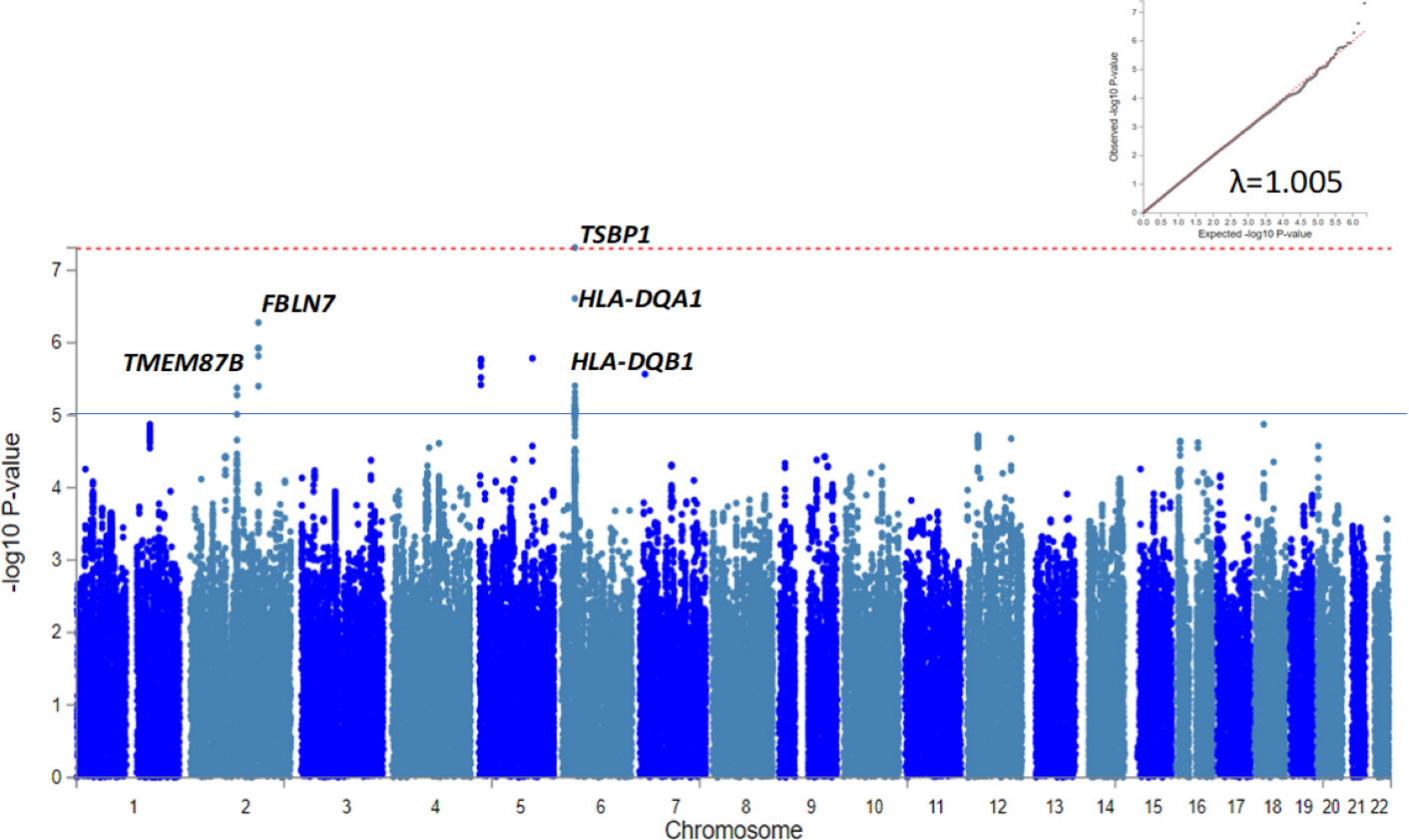
Manhattan plot for positive ANA in individuals of European ancestry. Loci associated with positive ANA at p≤5×10^−5^ are depicted. Red dotted and blue solid lines represent p≤5×10^−8^ and p≤1×10^−5^, respectively. Quantile-quantile plot (Q-Q plot) for p value associations and the genomic inflation factor in the right corner suggest absence of population stratification.

There were 5079 individuals (1654 ANA+ and 3425 ANA−) in BioVU and 2208 individuals (515 ANA+ and 1693 ANA−) in eMERGE. In BioVU, ANA+ compared with to ANA− individuals were more likely to be female (68.4% vs 56.1%, p=2.2×10^−16^) and older (median (IQR) age was 53.0 (41.0, 64.8) vs 50.0 (37.0, 61.0), p=5.2×10^−9^). None of the 5 190 297 SNPs that passed QC reached genome-wide significance (p≤5.0×10^−8^) in either BioVU (online supplemental figure S1 and table S3) or eMERGE (online supplemental figure S2 and table S4). A total of 2169 ANA+ and 5118 ANA− individuals were included in the meta-analysis; only one variant upstream of *TSBP1* within the *HLA* locus on chromosome 6, rs1967688, was associated with ANA+ (p=4.8×10^−8^), and this SNP was significantly associated with SLE in European populations (p=1.6×10^−9^, online supplemental table S5). Seven additional SNPs were associated with ANA+ at p≤1.0×10^−5^ (figure 2), but none of them were associated with SLE at p≤5×10^−8^. Three of these eight SNPs located in the *HLA* locus affect the expression of several HLA genes, and one SNP in chromosome 2 affects the expression of *TMEM87B* in blood. The genomic inflation factor (λ=1.005) suggested negligible population stratification.

### SNP-based heritability of ANA+ and PRS_ANA+_

The SNP-based heritability (h^2^_SNP_) of ANA+ was 0.04 (SD=0.07, p>0.05), thus genome-wide genetic correlation could not be performed. A PRS_ANA+_ derived from a meta-analysis including the training sample and eMERGE was not different in the ANA+ and ANA− individuals from the testing sample (p=0.98, online supplemental figure S3); thus, the PRS_ANA+_ was not analysed further.

### Associations between ANA+ and SLE-related SNPs and PRS_SLE_

**Figure 3:**
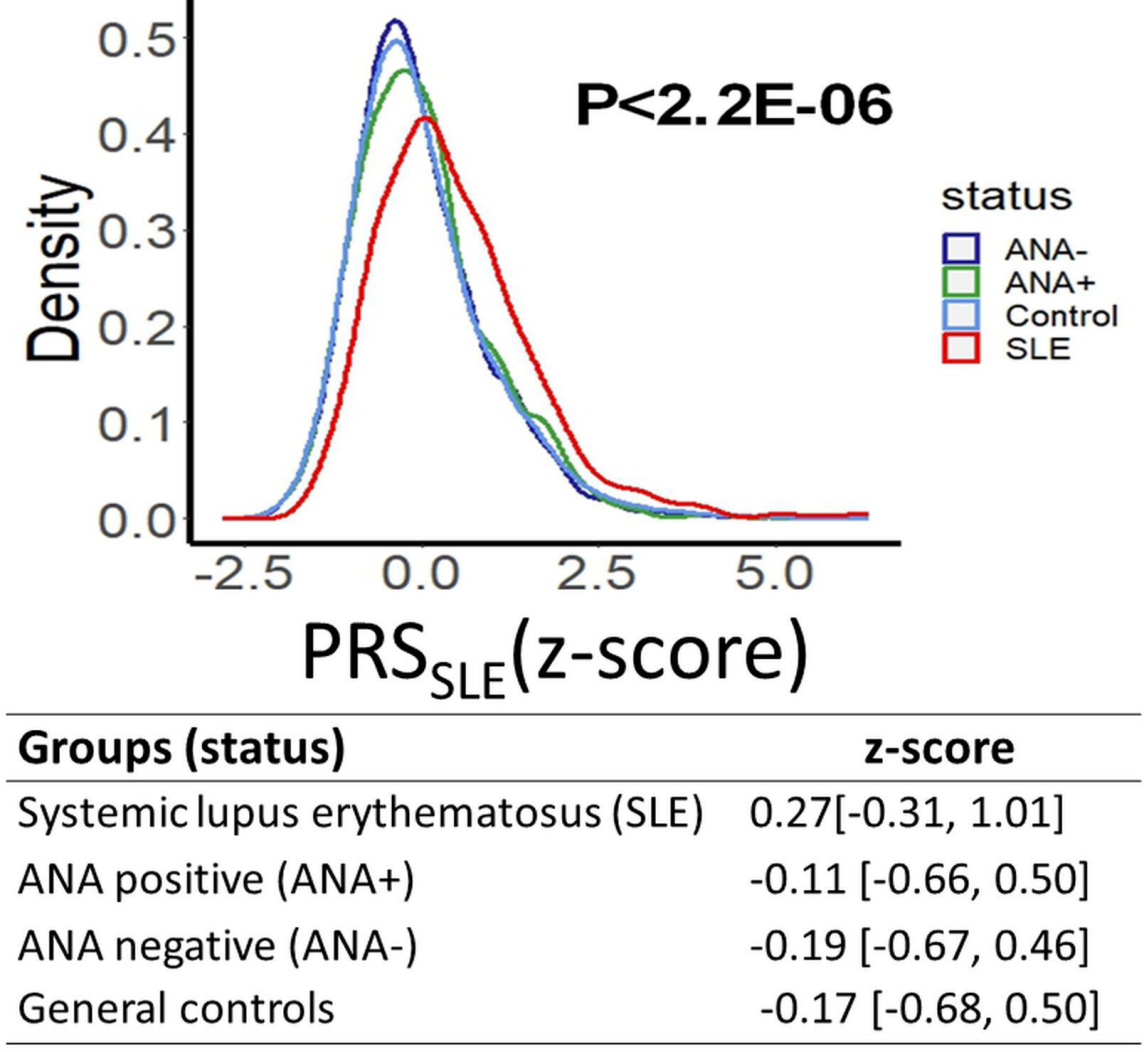
Distribution of the standardised polygenic risk score for SLE (PRS_SLE_ z-score) in patients with SLE, in individuals with a positive and negative ANA test and in controls. Patients with SLE had a higher standardised score (z-score) compared with the other three groups (p<2.2×10^−16^), and when comparing patients with SLE with ANA+ and ANA− individuals separately (p≤2.2×10^−16^ for both comparisons; data not shown). The PRS_SLE_ in controls was not significantly different from ANA+ and ANA− individuals (p=0.17).

Among the 33 autosomal SNPs associated with SLE that were available in the meta-analyses (online supplemental table S6), only rs11889341 (an intronic variant in *STAT4*) was significantly associated with ANA+ after correction for multiple comparisons (p≤0.002, table 1). Eight of the non-reported SNPs failed QC (three SNPs have an allele frequency below 5%, and five were not in HWE). In BioVU, a standardised PRS_SLE_ was significantly higher in patients with SLE (n=662) (median (IQR) 0.27 (−0.31, 1.01)) compared with ANA+ individuals without autoimmune disease (−0.11 (−0.66, 0.50); p<2×10^−16^), ANA− individuals (−0.19 (−0.67, 0.46); p<2×10^−16^) and general controls (n=65 374) (−0.17 (−0.68, 0.50); p<2.2×10^−16^, figure 3). The standardised PRS_SLE_ did not differ significantly among ANA+, ANA− and general controls without autoimmune disease (p=0.17).

**Table 1:** Association between SNPs significantly associated with SLE^13^ with positive ANA in individuals without an autoimmune disease

SNP	Chromosome	Location(GRCh37)	Locus	SLE-GWAS meta-analysis			ANA meta-analysis		
				RA	Estimate	P value	RA	Weighted z-score	WeightedP value
rs2476601	1	114 377 568	*PTPN22*	A	0.155	1.10E28	A	0.761	0.447
rs1801274	1	161 479 745	*FCGR2A*	G	0.064	1.04E-12	A	1.689	0.093
rs704840	1	173 226 195	*TNFSF4*	G	0.086	3.12E-19	G	2.426	0.015
rs17849501	1	183 542 323	*SMG7, NCF2*	T	0.322	3.45E-88			
rs3024505	1	206 939 904	*IL10*	A	0.068	4.64E-09	A	1.689	0.091
rs9782955*	1	236 039 877	*LYST*	C	0.064	1.25E-09	G	−0.009	0.993
rs6740462	2	65 667 272	*SPRED2*	A	0.041	2.67E-05	C	−1.64	0.101
rs2111485	2	163 110 536	*IFIH1*	G	0.061	1.27E-11	A	−2.317	0.021
rs11889341	2	191 943 742	*STAT4*	T	0.238	5.59E-122	T	3.217	0.001
rs3768792	2	213 871 709	*IKZF2*	G	0.093	1.21E-13	G	0.760	0.447
rs9311676	3	58 470 351	*ABHD6, PXK*	C	0.157	3.06E-14			
rs564799	3	159 728 987	*IL12A*	C	0.057	1.54E-09	T	−0.624	0.533
rs10028805	4	102 737 250	*BANK1*	G	0.079	4.31E-17	A	2.472	0.013
rs7726414	5	133 431 834	*TCF7, SKP1*	T	0.161	4.44E-16			
rs10036748	5	150 458 146	*TNIP1*	T	0.140	1.27E-45			
rs2431697	5	159 879 978	*MIR146A*	T	0.100	8.01E-28	C	0.181	0.857
rs1270942	6	31 918 860	*MHC class III*	G	0.358	2.25E-165	G	2.41	0.016
rs9462027	6	34 797 241	*UHRF1BP1*	A	0.057	7.55E-09	A	−0.576	0.565
rs6568431	6	106 588 806	*PRDM1, ATG5*	A	0.083	5.04E-14	A	0.960	0.337
rs6932056	6	138 242 437	*TNFAIP3*	C	0.262	1.97E-31			
rs849142	7	28 185 891	*JAZF1*	T	0.057	8.61E-11	C	0.827	0.408
rs4917014	7	50 305 863	*IKZF1*	T	0.072	6.39E-14	G	0.456	0.648
rs10488631	7	128 594 183	*IRF5*	C	0.283	9.37E-110	C	3.016	0.003
rs2736340	8	11 343 973	*BLK*	T	0.111	6.28E-20	T	0.427	0.670
rs2663052	10	50 069 395	*WDFY4*	G	0.064	5.25E-09	A	−0.505	0.613
rs4948496	10	63 805 617	*ARID5B*	C	0.057	1.04E-10	C	0.334	0.739
rs12802200	11	566 936	*IRF7*	C	0.090	8.81E-10	A	−1.495	0.135
rs2732549	11	35 088 399	*CD44*	A	0.093	1.20E-23	G	−1.588	0.112
rs3794060*	11	71 187 679	*DHCR7, NADSYN1*	C	0.090	1.32E-20	T	0.089	0.929
rs7941765	11	128 499 000	*ETS1, FLI1*	C	0.057	1.35E-10	T	−1.572	0.116
rs10774625	12	111 910 219	*SH2B3*	A	0.122	4.09E-09			
rs1059312	12	129 278 864	*SLC15A4*	G	0.068	1.48E-13	G	1.825	0.07
rs4902562	14	68 731 458	*RAD51B*	A	0.131	6.15E-10			
rs2289583	15	75 311 036	*CSK*	A	0.076	6.22E-15	A	1.798	0.072
rs9652601	16	11 174 365	*CIITA, SOCS1*	G	0.083	7.42E-17	A	0.570	0.568
rs34572943*	16	31 272 353	*ITGAM*	A	0.233	3.39E-76	C	−1.637	0.102
rs11644034	16	85 972 612	*IRF8*	G	0.097	9.58E-18	A	−2.91	0.004
rs2286672	17	4 712 617	*PLD2*	T	0.097	2.93E-09	T	0.097	0.922
rs2941509	17	37 921 194	*IKZF3*	T	0.130	7.98E-09			
rs2304256	19	10 475 652	*TYK2*	C	0.093	3.50E-13	A	−0.491	0.624
rs7444*	22	21 976 934	*UBE2L3*	C	0.104	1.84E-22	A	−0.717	0.473

* SNPs were not available in the meta-analyses results for ANA positive, and the table include association results with positive ANA for SNPs in linkage disequilibrium (r2≥0.8) in European populations: rs3768056(A) instead of rs9782955(C), rs28364617(T) instead of rs3794060(C), rs12917874(C) instead of rs34572943(A), and rs140489(A) instead of rs7444(C). Blank rows indicate SNPs that were not available in the ANA+ cohort. P≤0.0015 (0.05 corrected by 33 SNPs) were considered significant. Missing SNPs due to quality control procedures have minimal impact on the ANA positive with estimates and p value association ranging from 0.05 to 0.12 and 0.01 to 0.8, respectively.

AI, autoimmune disease; GWAS, genome-wide association studies; RA, risk allele; SNP, single nucleotide polymorphism.

## Discussion

This is the first study that analysed the genetic factors underlying a positive ANA test in individuals of European ancestry without autoimmune disease and their relationship with the genetic underpinnings of SLE. Our study showed a low estimated heritability for a positive ANA without autoimmune disease and only one shared SNP association with SLE.

Consistent with the role of HLA class II molecules in antigen presentation and antibody production, rs1967688 (the top risk allele) was associated with antibody seropositivity against *Staphylococcus aureus* in a Dutch population.^31^ This *HLA* SNP increased the risk of several autoimmune disorders associated with production of autoantibodies (eg, coeliac disease, type 1 diabetes, multiple sclerosis, rheumatoid arthritis) in PheWeb,^32^ suggesting its involvement in autoimmunity. Among the SNPs nominally associated with ANA+, rs9272346 (a SNP in the 5′ region upstream from *HLA-DQA1*) has been associated with the risk of asthma and type 1 diabetes.^33^ While rs12185740 has not been associated with any phenotype, the G allele increased the expression of several genes, including *MERTK* in blood, a gene implicated in immune tolerance that codes for a protein (MerTK) previously associated with disease activity and severity in SLE^34 35^ and with autoantibodies against chromatin and DNA in animal models.^36 37^

A previous GWAS for a positive ANA occurring in the absence of autoimmune disease performed in a Japanese population found that rs2395185, an intronic variant in the HLA region that is in modest LD with our top variant (r^2^=0.37 in European population), was associated with a positive ANA at a titre of 1:40 or higher. In this study, there was limited overlap with SLE risk loci (one non-HLA and one HLA SNP associated with SLE in Japanese and in European populations, respectively, were nominally associated with ANA+).^38^

While most common ANA-associated autoimmune diseases are highly heritable—with heritability estimates of 50% or more^39^,^44^—we found that the SNP heritability for ANA positivity in the absence of autoimmune disease was low (h^2^_SNP_=0.04). Accordingly, a PRS for ANA+ was not different in individuals with a positive and negative ANA test, suggesting that the role of genetic factors assessed by GWAS in ANA variability is modest and other factors such as environmental and epigenomic factors may be more important. This hypothesis is supported by previous findings from population and animal studies, where ANA positivity increases with age^45^ and with exposure to different drugs^46^ and chemicals.^47^,^53^

We found little evidence of overlap between the genetic factors associated with SLE and ANA positivity; we tested 33 SNPs significantly associated with SLE in previous GWAS studies, and only one was also significantly associated with ANA positivity. While several studies have consistently shown that the PRS for SLE is higher in patients with SLE compared with controls,^54^ this is the first time that the PRS for SLE was compared in patients with SLE and ANA+ individuals. We found that patients with SLE have a higher PRS compared with ANA+, ANA− and control individuals; moreover, the PRS for SLE did not differ significantly among the ANA+, ANA− and control groups. These findings suggest that a genetic predisposition to SLE is not associated with a predisposition to ANA+ in people without autoimmune disease.

The major strength of the study is the use of two cohorts of European ancestry that come from one of the largest clinical biobanks in the USA (BioVU)^16^ and from a large network of leading medical institutions in the USA.^17 18 23^ Our study has some limitations: first, while we excluded individuals who had a diagnostic code for common autoimmune diseases, a small proportion of those with a positive ANA test may have had undiagnosed autoimmune disease or could have developed lupus (or other autoimmune disease) in the future.^55^ This would have biased towards a finding of genetic overlap between SLE and ANA positivity, something we did not observe. Second, it is possible that some individuals who are ANA negative may become positive later in life. Third, we had power to detect OR ≥1.6 for SNPs with an allele frequency of 5% or larger in our GWAS and meta-analysis; thus, it is possible that common SNPs with smaller effects or with lower frequencies were not detected. Fourth, because of the limited sample sizes of other ancestral groups, we only studied people of European ancestry. Fifth, the effects of several genetic factors—such as rare variants, non-additive effects and interactions—were not captured. Sixth, while individuals with an ANA titre of 1:40 have some level of ANA expression, including these individuals would have increased the noise in the analyses and diluted genetic associations associated with higher ANA titres.

In summary, ANA positivity occurring in the absence of autoimmune disease has a genetic association with the HLA region, but overall heritability is low, suggesting an important role for non-genetic factors or genetic variation not captured in the GWAS. In addition, few SLE-associated SNPs were associated with ANA+, and the PRS for SLE was not associated with ANA+, indicating limited genetic overlap.

## Supplementary material

10.1136/lupus-2024-001476 online supplemental file 1

## Data Availability

Data are available in a public, open access repository.
