# Supplementary material for: Genetic relationships between systemic lupus erythematosus and a positive antinuclear antibody test in the absence of autoimmune disease
Source: Lupus Sci Med. 2025 Jun 12;12(1):e001476. doi: 10.1136/lupus-2024-001476 (PMC12164615; doi:10.1136/lupus-2024-001476)
Supplement: online supplemental file 1 [file lupus-12-1-s001.pdf]

Supplementary Tables and Figures

Table S1: Diagnostic codes for common ANA related autoimmune diseases

Table S2: Characteristics of the BioVU study population

Table S3: Top independent associations for ANA positive in individuals without an autoimmune disease of European ancestry in BioVU

Table S4: Top independent associations for ANA positive in individuals without an autoimmune disease of European ancestry in eMERGE.

Table S5: Top independent associations in the meta-analysis for ANA positive in individuals without an autoimmune disease and their associations with systemic lupus erythematosus in individuals of European ancestry.

Table S6: Associations with ANA+ in BioVU and eMERGE for SLE associated SNPs

Figure S1: Manhattan plot for ANA positivity without an autoimmune disease in individuals of European ancestry from BioVU. Loci with a SNP associated with positive ANA at P-value$\leq 1x10^{-5}$ (blue horizontal line) are shown. Quantile-quantile plot (Q-Q plot) for P-value associations (left corner) suggests absence of population stratification

Figure S2: Manhattan plot for ANA positivity without an autoimmune disease in individuals of European ancestry from eMERGE. Loci with a SNP associated with positive ANA at P-value$\leq 1.x10^{-5}$ (blue horizontal line) are shown. Quantile-quantile plot (Q-Q plot) for P-value associations (left corner) suggests absence of population stratification

Figure S3: Distribution of the standardized polygenic risk score positive ANA (PRS_{ANA+} z-score) in individuals with a positive and negative antinuclear antibody (ANA) test in the testing set. The PRS was constructed using meta-analysis results from the GWAS in BioVU (70% of the sample) and the eMERGE. The standardized score (z-score) was similar between ANA+ and ANA- individuals in the testing set (P-value=0.982)

Table S1: Diagnostic codes for common ANA related autoimmune diseases

ICD9	ICD10	Autoimmune disorder
373.34, 695.4, 695.4*, 710.0	H01.12*, L93, L93.*, M32, M32.0, M32.**	Systemic Lupus Erythematosus, Cutaneous Lupus
710.2	M35.0, M35.0*	Sjogren's disease
710.8, 710.9	M35.9, M35.5, M35.1, M35.8, M36.8	Unspecified diffuse connective tissue disease, Other specified diffuse diseases of connective tissue
517.2, 701.0, 710, 710.1, 710.3, 710.4,	M33, M33.*, M33.**, M34, M34.*, M34.**, M36.0, L94.*	Diffuse disease of connective tissue, Polymyositis, Dermatomyositis, Systemic sclerosis, Circumscribed scleroderma
571.42, 571.6	K75.4, K74.3	Autoimmune hepatitis, Primary biliary cirrhosis
714, 714.*, 714.**	M05, M05.*, M05.**, M05.***, M06.*, M06.**, M06.***, M08, M08.*, M08.**, M08.***	Rheumatoid arthritis and other inflammatory polyarthropathies: Rheumatoid arthritis, Juvenile rheumatoid arthritis
555, 555.*, 556, 556.**	K50, K50.*, K50.**, K50.***, K51, K51.*, K51.**, K51.***	Inflammatory bowel disease: Chron's disease, Ulcerative colitis
245.2	E06.3	Autoimmune thyroiditis
279, 279.*, 279.**, 283, 283.0	D59.1, D80.*, D81, D81.*, D82.* , D83, D83.*, D89.*	Others: autoimmune disease NEC, Other specified disorders involving the immune mechanism, Disorders involving the immune mechanism, Autoimmune hemolytic anemias

Table S2: Characteristics of the BioVU study population

Characteristics	ANA + (N=1653)	ANA – (n=3424)	P-value
Females (%)	1131 (68.4%)	1920 (56.1%)	2.2E-16
Age, years	53.0 [41.0, 51.5]	50.0 [37.0, 61.0]	5.2E-09
ANA Pattern	N= 416 (25.2%)	N=0	
Smooth	285 (17.2%)	Not applicable	Not applicable
Speckled	95 (5.7%)	Not applicable	Not applicable
Nucleolar	24 (1.5%)	Not applicable	Not applicable
Atypical	7 (0.4%)	Not applicable	Not applicable
Centromere	5 (0.3%)	Not applicable	Not applicable

Table S3: Top independent associations for ANA positive in individuals without an autoimmune disease of European ancestry in BioVU

SNP	Chr	bp (GRCh37)	RA	OA	estimate	SE	GWAS P-value	position	distance	closest gene
rs270182	5	5124875	A	G	-0.195	0.043	7.56E-06	intergenic	1283	*RN7SKP73*
rs6868392	5	24865888	G	A	0.194	0.043	5.38E-06	intergenic	16163	*RP11-730N24.2*
rs9275164	6	32652929	C	T	0.241	0.050	1.21E-06	intergenic	16768	*HLA-DQB1*
rs35084046	9	14758685	TA	T	0.233	0.046	4.18E-07	intronic	0	*FREM1*
rs112862863	12	99146042	CT	T	0.202	0.044	4.09E-06	intronic	0	*ANKS1B*
rs61692027	16	52441273	C	T	-0.366	0.079	4.00E-06	intergenic	30643	*TOX3*
rs141020039	16	65888976	G	A	-0.355	0.079	7.83E-06	intergenic	6038	*RP11-513N24.1*

SNP: single nucleotide polymorphism; Chr: chromosome; bp: base pair; RA: risk allele; OA: other allele; SE: standard error;

Table S4: Top independent associations for ANA positive in individuals without an autoimmune disease of European ancestry in eMERGE

SNP	Chr	bp (GRCh37)	RA	OA	estimate	SE	GWAS P-value	position	distance	closest gene
rs6684032	1	213104315	A	T	0.699	0.155	9.50E-06	intergenic	19546	*VASH2*
rs74148963	1	239308926	T	C	0.480	0.102	3.10E-06	intergenic	92723	*RP11-307O1.1*
rs2615328	2	164351418	T	A	0.546	0.110	1.89E-06	intergenic	98487	*FIGN*
rs76135609	2	217257791	G	T	0.631	0.137	7.10E-06	ncRNA_intronic	0	*AC098820.2*
rs13417769	2	218226173	G	A	0.491	0.104	3.99E-06	intronic	0	*DIRC3*
rs1352071	2	63357378	C	T	0.573	0.127	9.04E-06	intronic	0	*KCNH7*
rs35338326	2	217248274	G	A	0.623	0.137	8.48E-06	intergenic	1736	*AC098820.2*
rs113840449	10	53203986	A	G	1.126	0.240	5.31E-06	intronic	0	*PRKG1*
rs1426581	11	12584126	C	A	0.356	0.077	3.89E-06	intergenic	21612	*RP11-51B23.3*
rs2280481	12	53162206	T	A	-0.343	0.077	8.42E-06	UTR	0	*KRT76*
rs9533241	13	43350438	G	T	0.561	0.560	7.99E-07	intergenic	5247	FAM216B
rs77716437	14	91356462	G	A	-1.252	0.310	2.92E-06	intronic	0	*RPS6KA5*
rs74006953	15	27712431	G	A	0.933	0.190	2.72E-06	intronic	0	*GABRG3*
rs2442464	15	33711982	G	A	0.347	0.078	8.32E-06	intronic	0	*RYR3*
rs314233	17	7008258	A	G	0.366	0.080	9.18E-06	intronic	0	*ASGR2*
rs3760257	17	61496471	T	C	-0.427	0.097	7.19E-06	ncRNA_intronic	0	*TANC2*

SNP: single nucleotide polymorphism; Chr: chromosome; bp: base pair; RA: risk allele; OA: other allele; SE: standard error; UTR: untranslated region; nc: non-coding

Table S5: Top independent associations in the meta-analysis for ANA positive in individuals without an autoimmune disease and their associations with systemic lupus erythematosus in individuals of European ancestry

SNP	Chr	bp GRCh37)	RA	OA	Fixed beta	Fixed P-value	Q	I	Weighted z-score	Weighted P-value	SNP class	Near gene	eQTL in blood cells	SLE European population		
														A1	estimate	P-value
rs12185740	2	112889731	G	T	0.18	$3.7x10^{-6}$	0.82	0	4.6	$4.2x10^{-6}$	intergenic	*FBLN7*	*MERTK*	T	-0.06	0.05
rs2615328	2	164351418	T	A	0.03	$5.8x10^{-7}$	0.01	85.2	5.02	$5.3x10^{-7}$	intergenic	*FIGN*		A	-0.07	0.14
rs861512	5	5120776	G	A	-0.18	$1.3x10^{-6}$	0.59	0	-4.79	$1.7x10^{-6}$	intergenic	*RN7SKP73*		A	-0.01	0.67
rs6893553	5	128718653	T	C	0.31	$1.8x10^{-6}$	0.20	39.8	4.79	$1.6x10^{-6}$	intergenic	*MIR4460*		C	0.06	0.26
rs1967688	6	32340068	T	C	0.2	$8.2x10^{-8}$	0.07	69.9	5.46	$4.8x10^{-8}$	upstream	*TSBP1*	*HLA-DQA1, HLA-DQA2, HLA-DQB1, HLA-DQB1-AS1, HLA-DQB2, HLA-DRB1, HLA-DRB5, HLA-DRB6, HLA-DRB9, CA4*	C	0.18	$1.6x10^{-9}$
rs9272346	6	32604372	G	A	-0.17	$6.3x10^{-6}$	0.13	56.5	-4.62	$3.9x10^{-6}$	intronic	*HLA-DQA1*	*HLA-DQA1, HLA-DQA2, HLA-DQB1, HLA-DQB1-AS1, HLA-DQB2, HLA-DRB1, HLA-DRB5, TAP2*	A	-0.44	$2.3x10^{-4}$
rs9275140	6	32651018	A	G	0.50	$3.2x10^{-6}$	0.11	60.1	4.57	$4.8x10^{-6}$	intergenic	*HLA-DQB1*	*HLA-DQA1, HLA-DQA2, HLA-DQB1,* HLA-DQB1-AS1, *HLA-DQB2, HLA-DRB1, HLA-DRB6, HLA-DRB9*	G	-0.18	$3.5x10^{-7}$
rs117836487	7	13255078	A	T	0.40	$2.5x10^{-6}$	0.26	20.9	4.69	$2.7x10^{-6}$	ncRNA	*AC011288.2*		T	0.01	0.91

SNP: single nucleotide polymorphism; Chr: chromosome; bp: base pair; RA: risk allele; OA: other allele, Q and I: Cochran's Q and I^2 statistics that indicates probability of large variation across studies and the estimate variability across studies not due by chance, respectively; eQTL expression quantitative trait loci

Table S6: Associations with ANA+ in BioVU and eMERGE for SLE associated SNPs

SNP	Chr	bp (GRCh37)	SLE			BioVU					eMERGE				
			RA	OR	P-value	A1	A2	OR	SE	P-value	A1	A2	OR	SE	P-value
rs2476601	1	114377568	A	1.17	1.10E-28	A	G	1.05	0.07	0.505	A	G	1.05	0.13	0.710
rs1801274	1	161479745	G	1.07	1.04E-12	A	G	1.06	0.04	0.168	A	G	1.08	0.08	0.338
rs704840	1	173226195	G	1.09	3.12E-19	G	T	1.09	0.05	0.052	G	T	1.13	0.08	0.143
rs17849501	1	183542323	T	1.38	3.45E-88	T	C	1.11	0.09	0.230					
rs3024505	1	206939904	A	1.07	4.64E-09	A	G	1.04	0.06	0.534	A	G	1.24	0.10	0.034
rs9782955*	1	236039877	C	1.07	1.25E-09	G	A	1.02	0.05	0.705	T	C	0.94	0.09	0.447
rs6740462	2	65667272	A	1.04	2.67E-05	G	A	0.96	0.05	0.414	C	A	0.85	0.09	0.082
rs2111485	2	163110536	G	1.06	1.27E-11	A	G	0.91	0.04	0.028	A	G	0.93	0.08	0.380
rs11889341	2	191943742	T	1.27	5.59E-122	T	C	1.16	0.05	0.002	T	C	1.12	0.09	0.218
rs3768792	2	213871709	G	1.10	1.21E-13	G	A	1.04	0.06	0.500	G	A	1.04	0.11	0.721
rs9311676	3	58470351	C	1.17	3.06E-14						T	C	1.02	0.08	0.778
rs564799	3	159728987	C	1.06	1.54E-09	T	C	0.95	0.04	0.281	T	C	1.04	0.08	0.616
rs10028805	4	102737250	G	1.08	4.31E-17	A	G	1.15	0.04	0.001	A	G	0.96	0.08	0.627
rs7726414	5	133431834	T	1.17	4.44E-16						T	C	1.14	0.18	0.483
rs10036748	5	150458146	T	1.15	1.27E-45						T	C	1.10	0.09	0.263
rs2431697	5	159879978	T	1.11	8.01E-28	C	T	1.00	0.04	0.958	C	T	1.03	0.08	0.683
rs1270942	6	31918860	G	1.43	2.25E-165	G	A	1.09	0.07	0.215	G	A	1.34	0.12	0.012
rs9462027	6	34797241	A	1.06	7.55E-09	A	G	0.97	0.05	0.488	A	G	1.00	0.08	0.997
rs6568431	6	106588806	A	1.09	5.04E-14	A	C	1.06	0.04	0.195	A	C	0.98	0.08	0.825
rs6932056	6	138242437	C	1.30	1.97E-31						C	T	1.77	0.20	0.005
rs849142	7	28185891	T	1.06	8.61E-11	C	T	1.04	0.04	0.332	C	T	1.00	0.07	0.975
rs4917014	7	50305863	T	1.07	6.39E-14	G	T	1.00	0.05	0.952	G	T	1.08	0.08	0.358
rs10488631	7	128594183	C	1.33	9.37E-110	C	T	1.18	0.07	0.011	C	T	1.22	0.12	0.103
rs2736340	8	11343973	T	1.12	6.28E-20	T	C	0.99	0.05	0.783	T	C	1.11	0.09	0.233
rs2663052	10	50069395	G	1.07	5.25E-09	A	G	0.98	0.04	0.688	G	A	1.02	0.08	0.757
rs4948496	10	63805617	C	1.06	1.04E-10	C	T	1.03	0.04	0.534	T	C	1.03	0.08	0.736
rs12802200	11	566936	C	1.09	8.81E-10	A	C	0.98	0.05	0.703	A	C	0.82	0.09	0.032
rs2732549	11	35088399	A	1.10	1.20E-23	G	A	0.96	0.04	0.330	G	A	0.90	0.08	0.159
rs3794060*	11	71187679	C	1.09	1.32E-20	T	G	0.94	0.05	0.245	C	T	1.18	0.09	0.061
rs7941765	11	128499000	C	1.06	1.35E-10	T	C	0.95	0.04	0.275	T	C	0.91	0.08	0.230
rs10774625	12	111910219	A	1.13	4.09E-09						A	G	0.91	0.08	0.215
rs1059312	12	129278864	G	1.07	1.48E-13	G	A	1.02	0.04	0.663	G	A	1.23	0.08	0.008
rs4902562	14	68731458	A	1.14	6.15E-10						A	G	0.94	0.08	0.467
rs2289583	15	75311036	A	1.08	6.22E-15	A	C	1.08	0.05	0.087	A	C	1.06	0.08	0.501
rs9652601	16	11174365	G	1.09	7.42E-17	A	G	1.03	0.05	0.593	A	G	1.02	0.08	0.822
rs34572943*	16	31272353	A	1.26	3.39E-76	C	T	0.93	0.07	0.253	A	G	0.87	0.11	0.210
rs11644034	16	85972612	G	1.10	9.58E-18	A	G	0.91	0.05	0.065	A	G	0.80	0.09	0.013
rs2286672	17	4712617	T	1.10	2.93E-09	T	C	0.98	0.08	0.796	T	C	1.08	0.14	0.569
rs2941509	17	37921194	T	1.14	7.98E-09						T	C	1.01	0.20	0.941
rs2304256	19	10475652	C	1.10	3.50E-13	A	C	0.95	0.05	0.321	A	C	1.05	0.08	0.539
rs7444*	22	21976934	C	1.11	1.84E-22	G	A	0.97	0.06	0.557	C	T	0.96	0.09	0.679

SNPs are derived from the largest GWAS meta-analysis in European population. Independent SNPs with P-value≤$5x10^{-8}$ were extracted. RA and OA represent the risk and other allele, respectively. OR refer to A1 in BioVU and eMERGE.*SNP in LD (r2>0.8) available in BioVU (rs3768056, rs28364617, rs12917874, rs131659). Dark shaded rows represent SNPs unavailable in BioVU and/or eMERGE

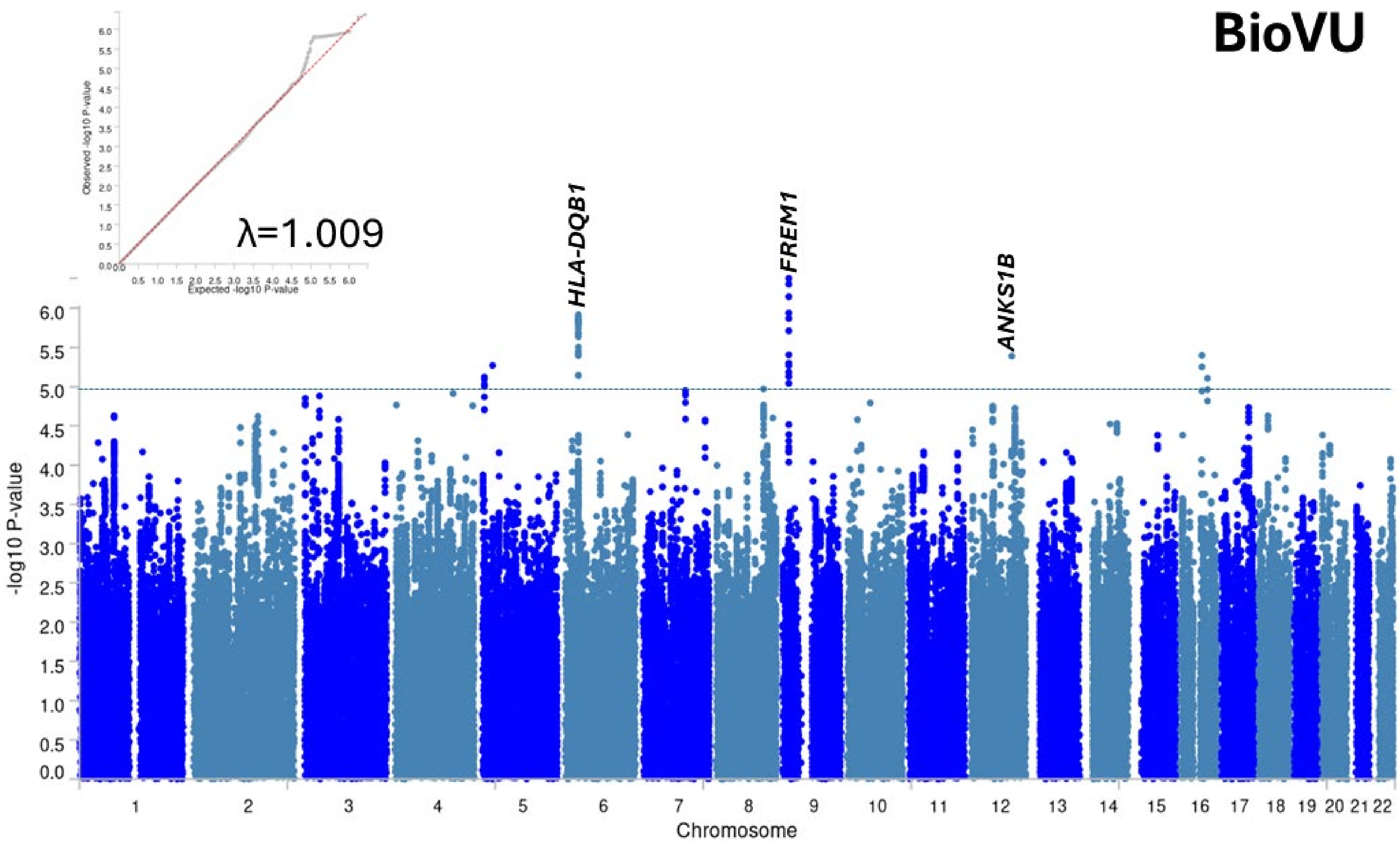

Figure S1: Manhattan plot for ANA positivity without an autoimmune disease in individuals of European ancestry from BioVU. Blue solid horizontal line represents P-value≤1×10^{-5} (blue horizontal line) are shown. Quantile-quantile plot (Q-Q plot) for P-value associations (left corner) suggests absence of population stratification

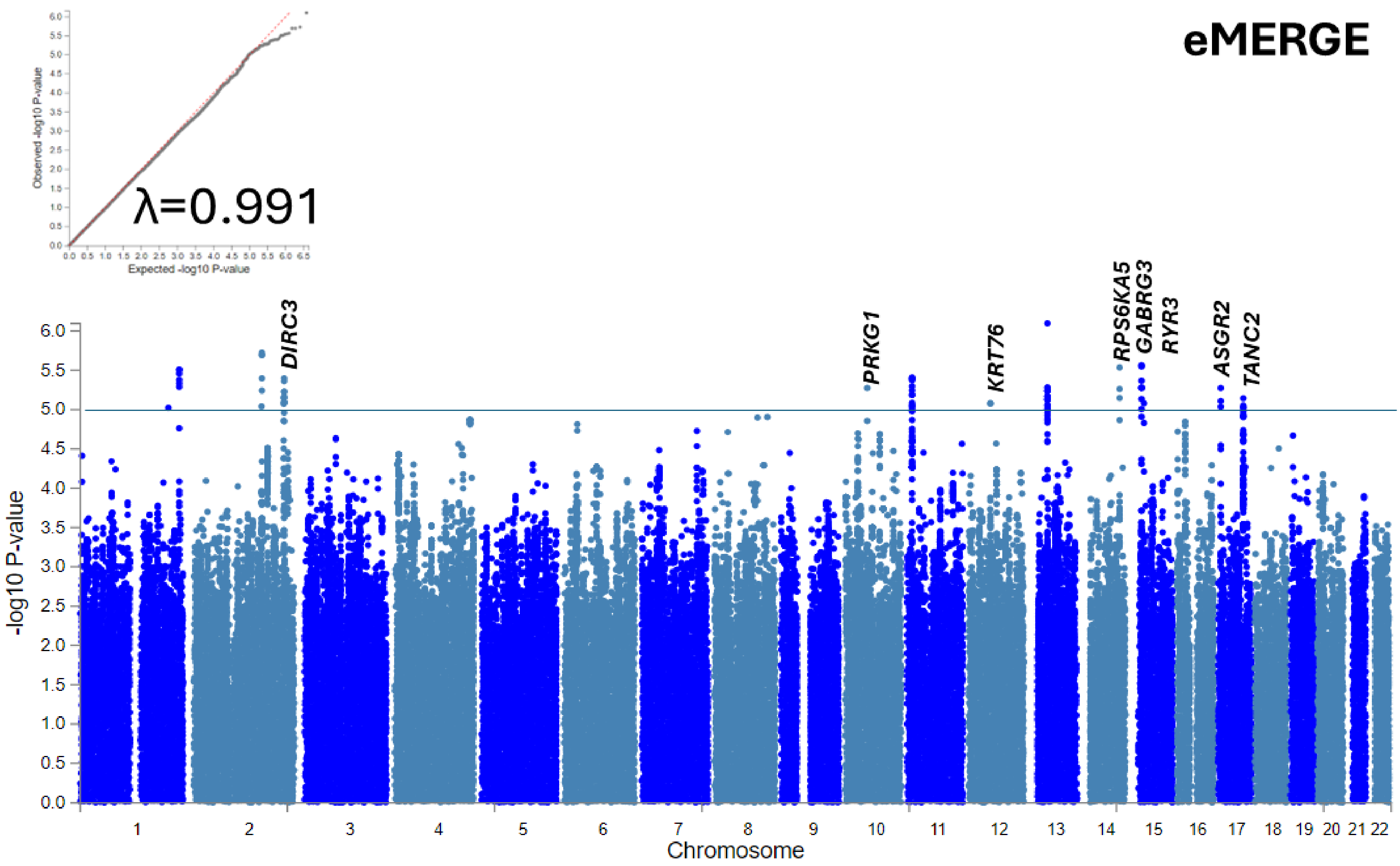

Figure S2: Manhattan plot for ANA positivity without an autoimmune disease in individuals of European ancestry from eMERGE. Blue solid horizontal line represents P≤1x10^{-5}. Quantile-quantile plot (Q-Q plot) for P-values associations (left corner) suggests absence of population stratification.

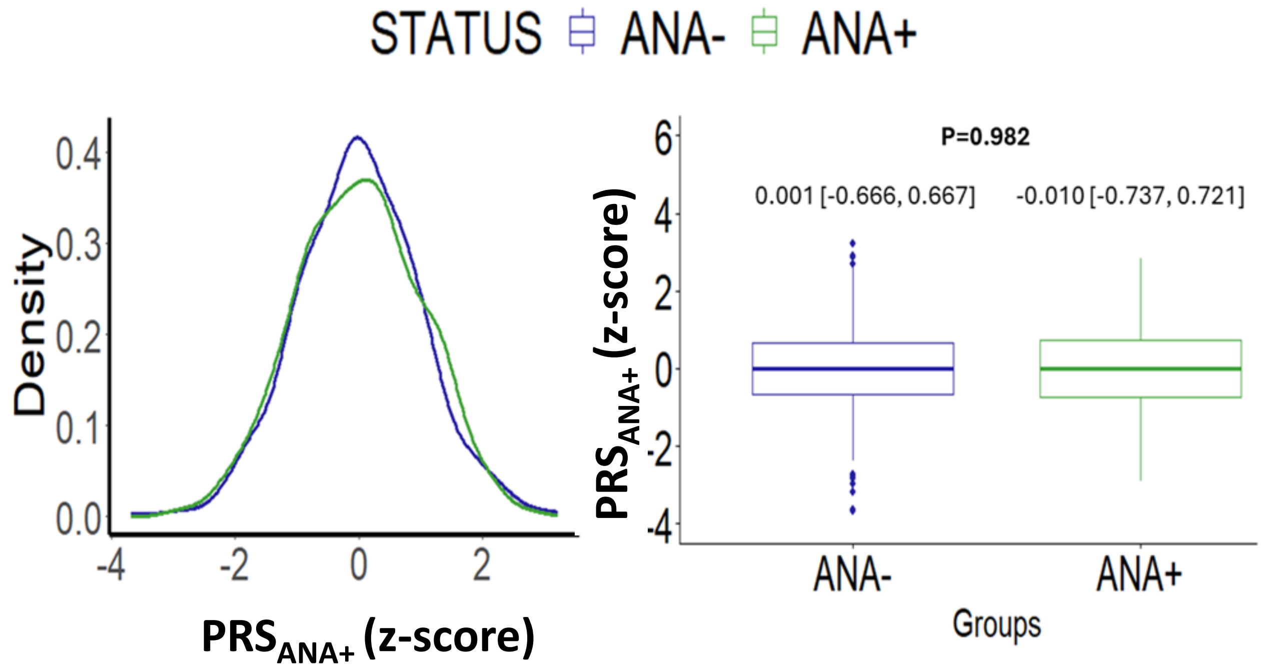

Figure S3: Distribution of the standardized polygenic risk score positive ANA (PRS_{ANA+} z-score) in individuals with a positive and negative antinuclear antibody (ANA) test in the testing set. The PRS was constructed using meta-analysis results from the GWAS in BioVU (70% of the sample) and in eMERGE. The standardized score (z-score) was similar between ANA+ and ANA- individuals in the testing set (P-value=0.982)
